# Mechanical Behavior of Crushed Waste Glass as Replacement of Aggregates

**DOI:** 10.3390/ma15228093

**Published:** 2022-11-15

**Authors:** Ali İhsan Çelik, Yasin Onuralp Özkılıç, Özer Zeybek, Memduh Karalar, Shaker Qaidi, Jawad Ahmad, Dumitru Doru Burduhos-Nergis, Costica Bejinariu

**Affiliations:** 1 Tomarza Mustafa Akincioglu Vocational School, Department of Construction, Kayseri University, Kayseri 38940, Turkey; 2 Department of Civil Engineering, Faculty of Engineering, Necmettin Erbakan University, Konya 42000, Turkey; 3 Department of Civil Engineering, Faculty of Engineering, Mugla Sitki Kocman University, Mugla 48000, Turkey; 4 Faculty of Engineering, Department of Civil Engineering, Zonguldak Bulent Ecevit University, Zonguldak 67100, Turkey; 5 Department of Civil Engineering, College of Engineering, University of Duhok, Duhok 42001, Iraq; 6 Department of Civil Engineering, College of Engineering, Nawroz University, Duhok 42001, Iraq; 7 Department of Civil Engineering, Military College of Engineering (Nust), Risalpur 24080, Pakistan; 8 Faculty of Materials Science and Engineering, Gheorghe Asachi Technical University of Iasi, 700050 Iasi, Romania

**Keywords:** eco-friendly concrete, waste glass, crushed, powder, hardened, fresh, slump, compressive, splitting, flexural, equation

## Abstract

In this study, ground glass powder and crushed waste glass were used to replace coarse and fine aggregates. Within the scope of the study, fine aggregate (FA) and coarse aggregate (CA) were changed separately with proportions of 10%, 20%, 40%, and 50%. According to the mechanical test, including compression, splitting tensile, and flexural tests, the waste glass powder creates a better pozzolanic effect and increases the strength, while the glass particles tend to decrease the strength when they are swapped with aggregates. As observed in the splitting tensile test, noteworthy progress in the tensile strength of the concrete was achieved by 14%, while the waste glass used as a fractional replacement for the fine aggregate. In samples where glass particles were swapped with CA, the tensile strength tended to decrease. It was noticed that with the adding of waste glass at 10%, 20%, 40%, and 50% of FA swapped, the increase in flexural strength was 3.2%, 6.3%, 11.1%, and 4.8%, respectively, in amount to the reference one (6.3 MPa). Scanning electron microscope (SEM) analysis consequences also confirm the strength consequences obtained from the experimental study. While it is seen that glass powder provides better bonding with cement with its pozzolanic effect and this has a positive effect on strength consequences, it is seen that voids are formed in the samples where large glass pieces are swapped with aggregate and this affects the strength negatively. Furthermore, simple equations using existing data in the literature and the consequences obtained from the current study were also developed to predict mechanical properties of the concrete with recycled glass for practical applications. Based on findings obtained from our study, 20% replacement for FA and CA with waste glass is recommended.

## 1. Introduction

In proportion to the increasing urbanization in the world, raw material resources are decreasing and industrial wastes are increasing [1,2,3,4,5,6,7,8,9,10,11]. This situation causes serious environmental problems. Scientists who are aware of the problem offer various construction element suggestions in order to consume fewer raw materials and to permanently evaluate the wastes by the construction industry. Recycling waste materials to utilize in the construction projects reduces the use and costs of raw materials, even when more than one ton of glass is recycled, 1.2 tons of raw materials can be saved [12]. In general, Europe’s glass recycling rate is 71.48%, while Slovenia and Belgium have a high rate of 98%. In Turkey, around 9% of glass waste can be recycled. While 52.9% of glass containers produced in the United States are disposed of in landfills, 26.63% of glass can be recycled [13]. Recycling glass as glass again takes time and is costly. Because it is subjected to processes at 1200–1400 °C, in order to get rid of wastes such as dirt and rust from the final product, it needs to be melted again and a product can be obtained [14]. Reusing waste glass in the production of glass provides 5–10% economic benefits [15]. However, adding it as aggregate to structural products, such as concrete, may provide more economic benefits [16,17,18]. Many recycled materials are used in concrete by replacing them with cement or aggregates [19,20,21]. Recycled crushed glass is used in numerous studies around the world as a substitute for CA and FA for the development of sustainable concrete [22,23,24]. One of the most important of these waste materials is crushed waste glass (CWG) in aggregate size. The use of broken glass particles by substituting aggregates in concrete production is an important and interesting issue. Since glass does not change its structure when reprocessed, its chemical properties do not deteriorate and it is very easy to reprocess. Some glass products have a limited lifespan depending on their intended use. This increases the potential of broken waste glass particles. This recycling potential needs to be processed quickly [25]. In addition, the degradation rate of CWG is lower than industrial products, such as plastic, paper, and rubber. Therefore, the usage of broken glass fragments as a substitute for aggregates in concrete production will yield impressive consequences [26].

In recent years, much research was conducted on the mechanical properties of CWG added concretes. The CWG can be substituted for both fine and CAs [27]. The nature of glass reactivity causes significant effects when added to concrete. For example, some may cause excessive expansion in concrete when used as 100% of the total aggregate [28]. Studies show that with CWG aggregates, as the rate of change increases, the strength decreases faster. For example, in a study that used 15−60% substitution, it decreased the CS by 8% with the addition of 15% CWG, while it decreased the CS by 15% with the addition of 30% CWG. When this proportion is increased to 60%, the CS decreases by 49% [29]. Singh and Siddique, in their study, by replacing 10–50% CWG with FA, found that the strength decreased as the CWG proportion increased. However, when CWG was used with methacholine, they discovered that the strength improved as the methacholine addition increased [26]. This result shows that additional reinforcing materials can be added to increase the mechanical properties of the concrete whose environmental impact is increased with CWG. Harrison et al. used fine glass particles by substituting cement and FA in certain proportions in hopes of benefiting the mechanical properties of concrete. According to the consequences obtained, the cement preserves its mechanical properties when CWG particles are added up to 20%, but when the additions of 30% and above cause insufficient CaCO_3_ in the cement, the mechanical properties are adversely affected. It was also found that with the change in FA conservative, its mechanical properties are up to 20% substitution [27]. It was confirmed in some studies that the CS of concrete increases as the size of CWG particles is substituted with aggregate decreases. In their study, Shi et al. observed that finer glass particles improved the 28-day CS of concrete more than larger ones [30]. Chen et al., on the other hand, found that finer-ground glass particles performed better with similar behavior in 7-day and 28-day concrete CS [31]. Khmiri’s et al. found that with up to 20% cement replacement, CWG ground up to 20 µm improved the late mechanical properties of concrete, while 40 µm CWG reduced the CS [32]. Letelier and Al-Hashmi found that 38 µm CWG had optimal mechanical properties at 20% cement substitute (by weight). In another study, it was revealed that 38 µm CWG had optimal mechanical properties at 20% cement replacement (by weight). It was determined that 10% and 30% substitutions gave consequences close to the reference sample [33]. Mostofinejad et al., examining 30% of cement by weight for ground CWG, found that the replacement had a strength reduction of 40% and 42%, respectively [34]. Tamanna et al. used 3000 µm CWG by replacing 20%, 40%, and 60% coarse sand. As a result of the 7, 28, and 56-day compressive strength (CS) and flexural strength (FS) tests, they discovered that the 20% substitution gave good consequences, while the 40% and 60% substitution had negative effects on the CS and FS [35]. Penacho et al. investigated the effects of replacing 20%, 50%, and 100% CWG with FA on the mechanical properties of concrete. As a result of the analysis, he found that samples with higher sand substitution for fine CWG gave a greater strength increase. The increase in strength is related to the positive effect of pozzolanic reaction with the fine glass particles [36]. Lee et al. investigated the size effect of glass waste on the mechanical properties of concrete. As a result of the research, they found that the CS was better in samples consisting of particles smaller than 600 µm [37]. Corinaldesi et al. performed tests by replacing glasses with glass particles smaller than 36 µm, 36–50 µm, and 50–100 µm with aggregate. They performed compressive and FS tests at 180 days to study the influence of particle size. As a result of the observation, they found that the CS decreased at 30% FA substitution. However, at the 70% substitution proportion, they found that 50–100 µm samples showed milder increases over the reference sample [38]. Tejaswi et al. In a study they conducted, they found that replacing 20% by weight of CWG with FA gave a result close to the control sample, but replacing 10%, 30%, 40%, and 50% lowered the CS [39]. Batayneh et al. reported that replacing the CWG with the addition of fly ash increased the CS; however, they revealed that it did not change the splitting strength [40]. With a similar statement, Gerges et al. found that the rate of substitution of fine glass with sand had little effect on the compressive, splitting stress, or flexural strengths of concrete [41]. As a result of their study, Mohammed and Hama determined that when glass is added to concrete alone, it improves properties, such as CS, FS, and splitting tensile strength (STS). Compared to the reference sample, an increase of 14.12%, 1.7%, 6.01%, 52.63%, and 57.32% was observed in elastic modulus, energy capacity, and bond strength, respectively [42]. In a study by, Asa et al., it was found that the concrete to which 5% and 20% glass fragments were added led to a decrease of 3.8–10.6 percent and 3.9–16.4 percent, respectively, in the CS and tensile strength at the end of the 21st day, but the use of mineral additives changed the properties of the mixtures. They found that it improved after 7, 14, and 21 days of testing [25]. Walczak et al. used cathode ray tubes glass instead of sand, and they found that the compression strength increased about 16% and the bending strength increased about 14% [43].

Though several investigations were performed on this topic, as presented in above, there were changes in the consequences gained from the literature. Therefore, there is still a necessity to examine the mechanical productivity of concrete with the fractional replacement of CWG, and the perfect amount of it. For this purpose, an investigational study was performed on some investigation samples. The effects of different production-based features of concrete with different amounts of crushed CWG as replacement of aggregates were studied. More importantly is that empirical equations are developed to predict the capacity of concrete with CWG, considering both the literature and the data obtained from the experimental study.

## 2. Experimental Program

To amount the mechanical belongings of the concrete with recycled CWG, different mixtures were cast. Eight different mixtures were chosen. Four of them were considered for FAs and rest of them were considered for CAs. Four different proportions of 10%, 20%, 40%, and 50% were selected (Table 1). CAs with a size of 5–13 mm were utilized while FAs with sizes of 0–4 mm were utilized. FA represents FA replacement and CA represents CA replacement. CWG was collected from Akcihan Glass, Istanbul, Turkey, involving a waste window glass (soda lime glass). The size of fine glass is a combination of glass with an equal amount of 1.7–4 mm and 100–200 micron, while the size of coarse glass is a combination of glass with an equal amount of 9–12 mm and 5–8 mm.

**Figure 1:**
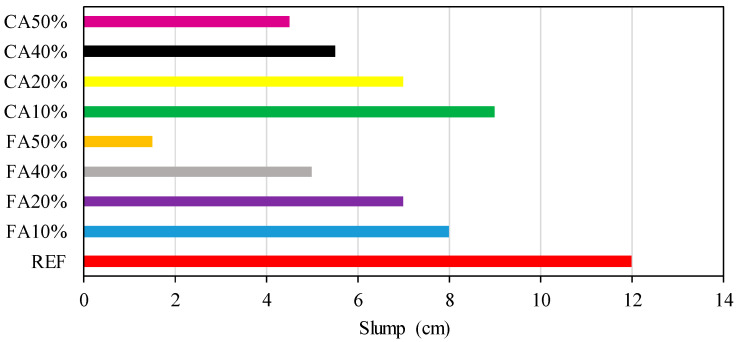
Slump test.

In the mixture, CEM 32.5 Portland cement was utilized. Water-to-cement proportion was selected as 0.5. The proportion of fine to CAs was selected as unity. The proportion of cement to total fine and CAs was selected as 0.2. Figure 1 shows slump test consequences of each mixture. The highest slump value was observed in the reference mixture. Adding recycled glass in concrete reduced slump value. Replacing aggregate resulted in a significant decrease in workability, especially after 20%. This is because glass particles have sharper and irregular geometric forms than sand particles, which can cause high friction, resulting in less fluidity [44]. Moreover, the binder effect of glass powder in FA replacement reduced workability even more than replacement of CA.

## 3. Experimental Consequences and Discussions

### 3.1. Compressive Strength (CS)

**Figure 2:**
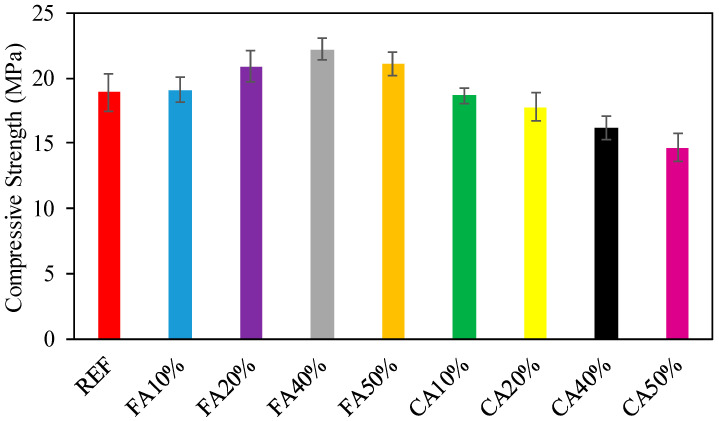
Consequences of CS.

Figure 2 demonstrates the CS test consequences at a 28-day period formed with 100% reference and numerous proportions of CWG of fine and CA exchanging reference aggregates. As recognized in Figure 2, in the midst of the low CWG proportion, the CSs of concrete mixtures with CWG consumption as a replacement for FA were greater than those of the consistent concrete mixes lacking CWG. As presented in Figure 2, fine and CA for 10%, 20%, 40%, and 50% (FA10%, FA20%, FA40%, FA50%, and CA10%, CA20%, CA40%, and CA50%) were exchanged with CWG; this inclination was reversed while fractional replacement of CA with CWG decreased the CS of concrete. Statistical examination of specimen consequences indicated the important proceeds of CWG (as FA was swapped with CWG) to the CS of concrete. As the evaluation of the FA is swapped with CWG powder, the CS of FA10% (10% FA were swapped with CWG) is 1% greater than that of reference concrete. Similarly, the CS of FA20% (20% FA were swapped with CWG) is 9% greater than that of FA10%. At the evaluation of CS of FA40% (40% FA were swapped with CWG) is 6.2% larger than that of FA20%. Lastly, at the evaluation of CS of FA50% (50% FA were swapped with CWG) is 5.2% greater than that of FA40%. While compared with reference concrete, the CS of FA50% (50% FA were swapped with CWG) is comparable with that of reference concrete. The evaluation of CS of FA50% (50% FA were swapped with CWG) is 12% greater than that of reference concrete. The increase in the capacity with FA can be explained with the use of glass powder since the glass powder has a binder effect. Alternatively, at the evaluation of CS of CA10%, CA20%, CA40%, and CA50% (CAs were swapped with CWG), the CS test consequences for the great CWG proportion downgraded trends comparable to those for the small CWG proportion. In this case too, the CS of CA10% is 27% larger than the corresponding mix (CA50%). While CA was swapped with CWG, a remarkable downgrade in strength was detected when compared with the reference concrete and FA50%. This decrease in CS can be explained by two reasons. One of the reasons is that glass waste has a smooth surface compared to normal aggregates, which causes a decrease in the bond among the particles and the cement matrix, and the decrease in CS increases as the glass percentage increases. The second reason is that the absorption of CWG is less than normal aggregates, and this causes higher slump values. For this reason, there is an extra amount of free water that evaporates, leaving a little more space, which causes a decrease in CS [45].

### 3.2. Splitting Tensile Strength (STS)

**Figure 3:**
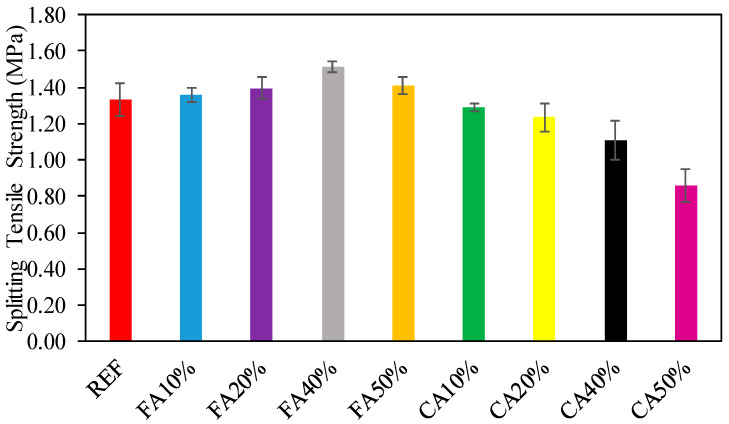
Consequences of STS.

**Figure 4:**
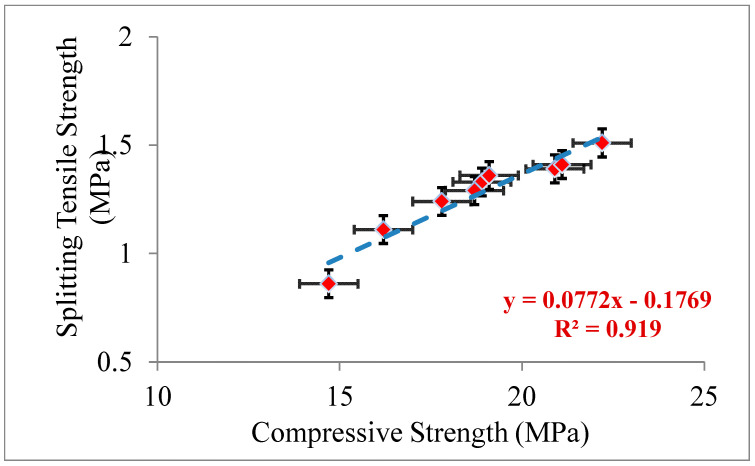
Splitting tensile strength variation versus compressive strength.

An evaluation of the relation STS of the numerous concrete proportion mixtures made with the reference, and several proportions of recycled aggregate replacing FA with and without CWG and CA, which were swapped with CWG, is presented in Figure 3. As presented in Figure 3, the consequences of STS generally follow a similar trend as the CS. As detected from Figure 3, the significant progress in STS of concrete is up to 13%, with CWG used as fractional replacement for FA. The reason for this can be shown as the progress of hydration, decreased permeability of glass-mixed concrete, good bond strength among the glass aggregate, and the surrounding cement paste due to the irregular geometry of the glass. The pozzolanic reaction may also offset this trend at a later stage of hardening and help improve the STS at 28 days [44]. Upon changing the amount of CWG, outcomes of tensile strength are affected correspondingly to the CA. This decrease in strength can be explained due to the surface structure of CWG. Similar results were also observed by others [45,46,47]. As presented in Figure 3, tensile strength optimum values are established at 40% CWG, requiring maximum distributed tensile. The correlation of CS in competition with STS is provided in Figure 4. As presented in Figure 4, the regression model among the CS and STS is performed to be flat. Additionally, as detected in Figure 4, the regression stroke characterizes a strong relationship among CS, compared with STS needing an R^2^ worth of more than 92%.

### 3.3. Flexural Strength (FS)

**Figure 5:**
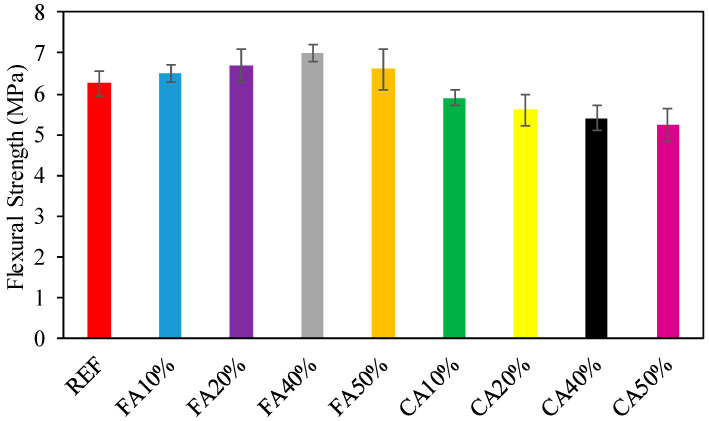
Consequences of FS.

**Figure 6:**
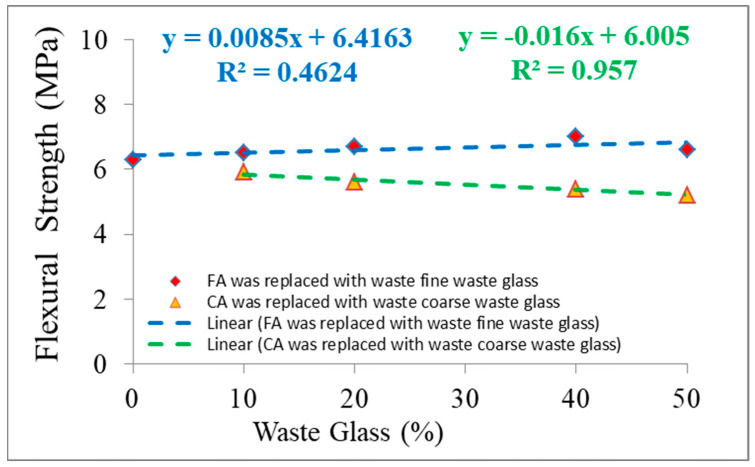
Consequences of tensile FS tests of concrete examples with changed CWG substances.

FS of investigational specimens were recognized on specimens after CST. The assimilated types of strength alternated among 5.2 MPa and 7.0 MPa. The result of the FS of the specimens is offered in Figure 5. In Figure 5, FS with different proportions of CWG is shown. It was noticed that with the addition of CWG at 10%, 20%, 40%, and 50% of FA swapped, the increase in FS was 3.2%, 6.3%, and 11.1% and 4.8%, respectively, in percentage to the reference sample (6.3 MPa). This could be dedicated to pozzolanic reactions, which accelerate with time, and offset the hardening process and aid the increase in FS [44]. A related behavior was also described by Shehata et al. [48]. It must be noted that higher CWG replacement may have an opposing effect on the FS [48]. Figure 6 shows a rectilinear relationship among the tensile FS of the example and the substance of the CWG addition. Alternatively, if FAs were swapped with CWG, it was observed that the increase in FS was 11.1%, respectively, in amount to the reference sample (6.3 MPa). However, if the amount of CWG is employed in relation to the CA contented, it may be noticed that the FS and CWG contented are associated (Figure 6)). This can be attributed to the change in the interfacial transition zone properties of the glass-containing mixtures [49]. Additionally, as detected in Figure 6, the regression shows a good relationship among FS contrasted with CWG having an R^2^ worth of more than 95%.

## 4. Scanning Electron Microscope (SEM) Analysis

**Figure 7:**
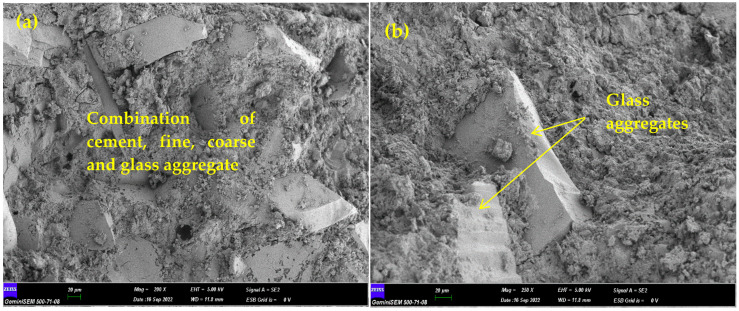

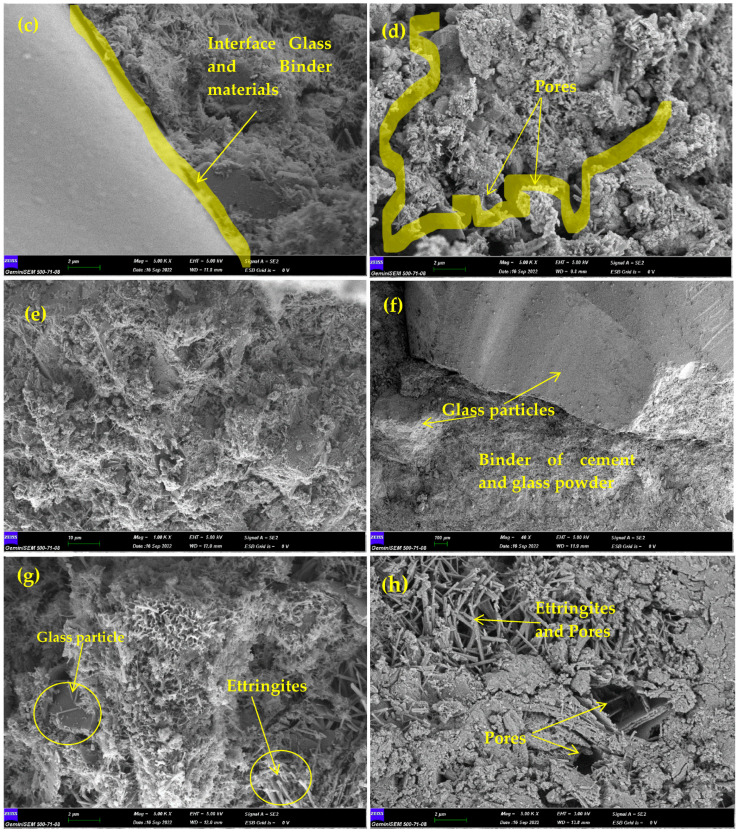
Consequences of SEM analysis.

SEM investigation was achieved with samples taken from some samples of this experimental study. The images are magnified 500 times to show the interaction among binder materials and aggregates. In SEM analysis, there are images of two different mixtures. The first four are images of concrete samples containing only CGW, and the last four are images of samples containing both CGW and glass powder. Figure 7a shows the situation where CGW is swapped by aggregate. It is seen that the glass pieces, in the range of 4–9 mm, are homogeneously dispersed in the homogeneous concrete by providing good compatibility with the cement binder. Figure 7b shows the slightly larger and closer state glass particles dispersed in the concrete and their harmonious positions. In Figure 7c, it can be said that the interface among the glass piece and the binding cement remains in a discrete form. Figure 7d shows that gaps and holes are formed in some regions. This problem can be eliminated with better compression and vibration. Glass powder makes a good pozzolanic effect because it has a higher specific surface area than cement [30,50]. It can be seen in Figure 7e that the binding property of the CWG powder cement and the pozzolanic property of the glass powder results in good bonding. Etringite formation is an important issue in Portland cement concretes due to early phase hydration [51,52]. Although the hydration of glass powder and cement show similarities, some ettringite formation is seen in Figure 7e. While the samples were being prepared, a good mixing in the mixer ensured the homogeneous distribution of the additional powder particles. Although a very small part was examined in the SEM analysis, the compressive and FS consequences show a homogeneous distribution among homogeneous binders with an increasing productivity in the range of 10–40%. Figure 7f shows the standing of small and large CGWs in concrete. While there is a line among the large particle and the cement, it is seen that the smaller piece of glass has a better bonding with the cement. It is stated that the use of glass particles as aggregate can increase cracks and voids, and this will cause a decrease in strength [53]. Figure 7g shows the distribution of glass powders and small glass particles in the concrete. It can be said that the use of both CWG together for concrete is a good match. In Figure 7h, it is seen that there are gaps and holes in places. These can be considered as mini problems. It is stated in some studies that glass powder with finer particles has a high pozzolanic effect and provides better strength in concrete. The consequences obtained confirm the statements in the literature [53,54,55,56].

## 5. Evaluation of Current Findings with Previous Studies

**Figure 8:**
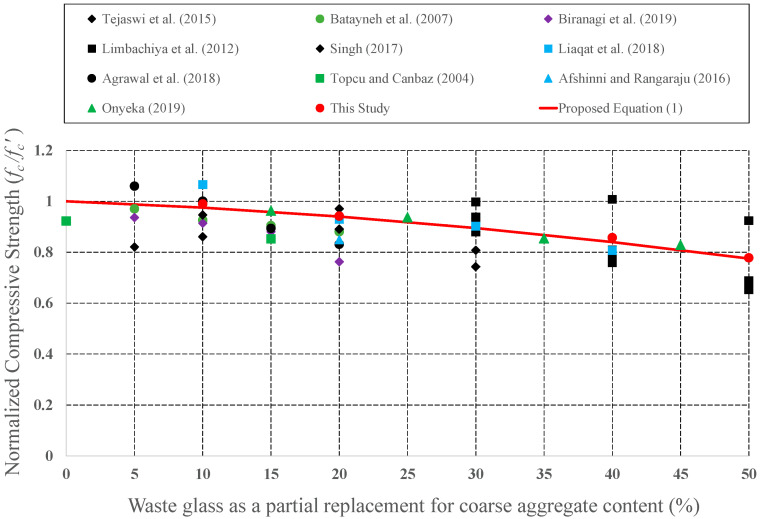
Discrepancy of the normalized CS of the concrete formed with CWG as a partial replacement for CA [39,40,47,58,59,60,61,62,78,79].

**Figure 9:**
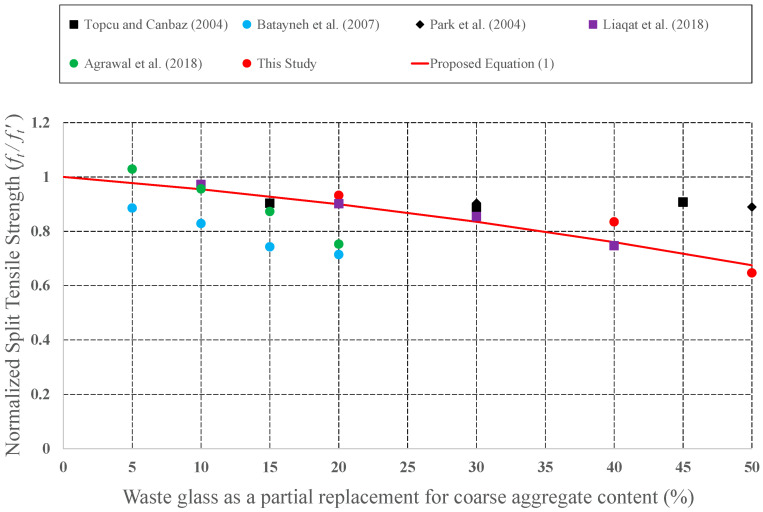
Discrepancy of the regulated STS of the concrete formed with CWG as a partial replacement for CA [40,46,47,60,79].

**Figure 10:**
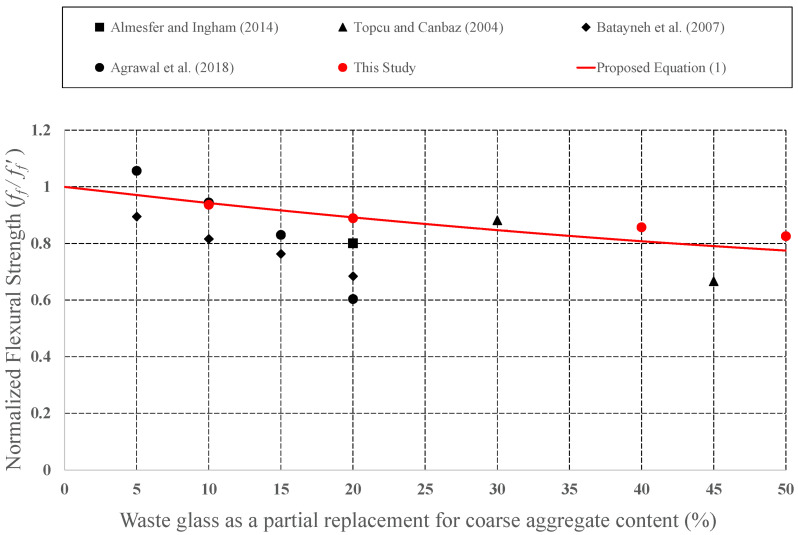
Discrepancy of the normalized FS of the concrete produced with CWG as a partial replacement for CA [40,47,57,79].

**Figure 11:**
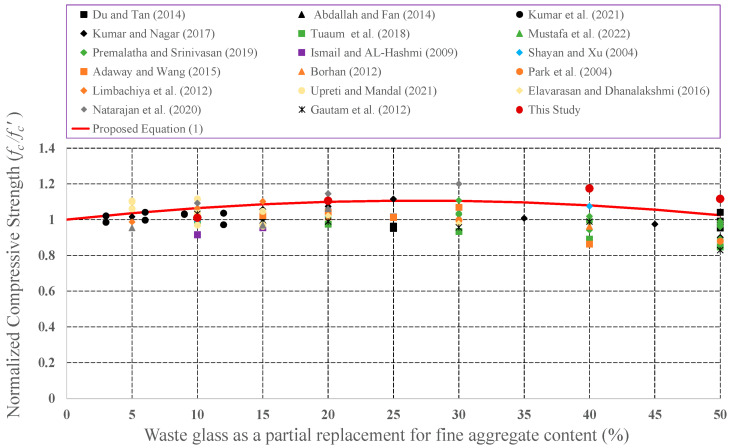
Discrepancy of the normalized CS of the concrete formed with CWG as a partial replacement for FA [28,33,44,46,58,63,64,65,66,67,69,70,71,72,73,74,77].

**Figure 12:**
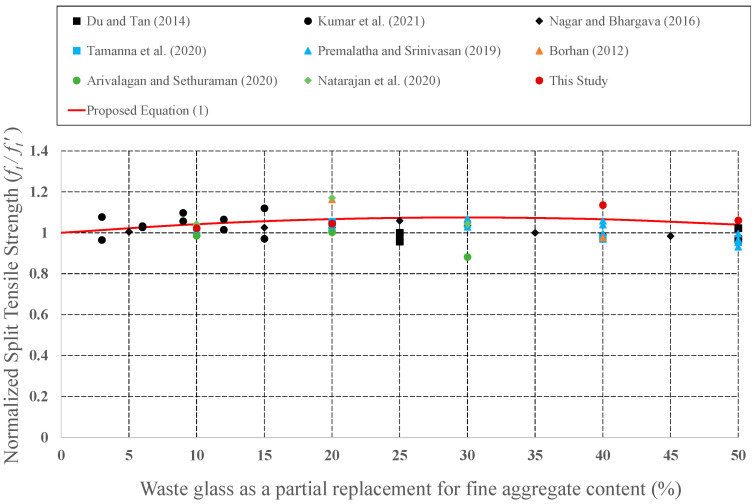
Discrepancy of the regulated Splitting tensile strength of the concrete produced with CWG as a partial replacement for FA [29,35,63,64,69,70,73,80].

**Figure 13:**
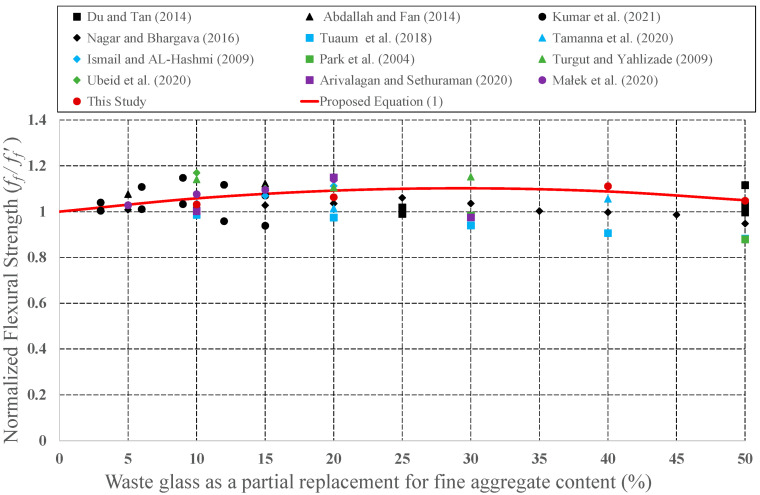
Discrepancy of the normalized FS of the concrete produced with CWG as a partial replacement for FA [29,33,35,44,46,63,64,66,68,75,76,80].

Since it has the potential to be preferred as aggregate in concrete mixture, the effect of CWG on engineering properties was examined by many investigators. CWG were used as either FA or CA replacement in concrete. To develop empirical equations, strength values (CS, STS, and FS) for plain concrete and concrete formed from CWG were collected from previous experimental studies [28,29,33,35,39,40,44,46,47,57,58,59,60,61,62,63,64,65,66,67,68,69,70,71,72,73,74,75,76,77,78,79,80]. The obtained strength values of concrete formed with CWG (*f*) were primary regularized by the strength value of concrete without glass (*f*′). These normalized strength values (*f***/***f*′) were then shown as a function of different replacement proportions. The changes in the normalized strength values were depicted in Figure 8, Figure 9, Figure 10, Figure 11, Figure 12 and Figure 13.

Considering both our findings and previous studies, an empirical equation was developed as follows to predict the CS, STS, and FS, respectively:

(1) $$f=\left[1+c_{1}\times \left(WGR\right)+c_{2}\times {\left(WGR\right)}^{2}\right]\times f^{\prime }$$

where *f* = strength values to be calculated (*f_c_* = CS; *f_s_* = STS; *f_f_* = FS); *c*_1_ and *c*_2_ = the coefficients given in Table 2; *WGR*: CWG proportion (0 < *WGR* < 50); and $f^{\prime }$ = strength values of the plain concrete.

As presented in Equation (1), engineering properties of concrete formed with CWG were identified as a function of the quantity of the CWG. The developed expressions for compressive, flexural, and splitting tensile strength of concrete produced with CWG can be employed in project phases.

## 6. Conclusions and Summary

In this study, the effects of different productions based features of concrete with different amounts of CWG as replacements of aggregates were investigated. For this purpose, fine and CAs were altered for 10%, 20%, 40%, and 50% (FA10%, FA20%, FA40%, FA50%, and CA10%, CA20%, CA40%, and CA50%). Penetrability and slump properties of produced concrete samples were also examined. Then, CS, STS, and FS of the produced test examples were investigated. Furthermore, SEM analyses were also performed to compare the strength consequences obtained from the experimental study. These specifications were then compared with those of reference concrete. Lastly, practical equations were derived to easily estimate the CS, STS, and FS of produced concrete samples. Considering our findings, the following consequences can be obtained from this study:

According to the slump test consequences, while the slump value reduces, the quantity of CWG rises. In other words, the workability of concrete reduces when the rate of glass powder as replacement for aggregates increases.

In this study, fine and CA for 10%, 20%, 40%, and 50% (FA10%, FA20%, FA40%, FA50%, and CA10%, CA20%, CA40%, and CA50%) were exchanged with CWG, and this tendency was inverted while fractional replacement of CA with CWG decrease the CS of concrete. In other words, while CA was swapped with CWG, a remarkable downgrade in strength was detected when compared with the reference concrete.

As mentioned above, the consequences of STS generally follow a similar trend as the CS. The significant progress in STS of the concrete is up to 13% with CWG used as fractional replacement for FA. Upon changing the quantity of CWG, consequences of STS are affected correspondingly to the CA.

While aggregates were swapped with CWG, there was an increase in the FS values up to a certain value of the quantity of the CWG. It was observed that with the addition of CWG at 10%, 20%, 40%, and 50% of FA swapped, the increase in FS was 3.2%, 6.3%, and 11.1% and 4.8%, respectively, compared to reference concrete sample.

From the SEM analyses, it can be observed that the use of both CWG together for concrete is a good match. Furthermore, similar to the studies in the literature, it is also shown in this study that glass powder with finer particles has a high pozzolanic effect and provides better strength in concrete.

The developed empirical equations for the CS, STS, and FS are quite general and they have the potential to be implemented into design guidelines of the concretes with CWG.

The use of 20% replacement for fine and CA with CWG is recommended, considering both workability and strength.

## Figures and Tables

**Table 1:** Different mixtures of the concrete with recycled glass.

REF. SAMPLE	Without Glass
CA10%	10% CA was swapped with waste coarse waste glass
CA20%	20% CA was swapped with waste coarse waste glass
CA40%	40% CA was swapped with waste coarse waste glass
CA50%	50% CA was swapped with waste coarse waste glass
FA10%	10% FA was swapped with waste fine waste glass
FA20%	20% FA was swapped with waste fine waste glass
FA40%	40% FA was swapped with waste fine waste glass
FA50%	50% FA was swapped with waste fine waste glass

**Table 2:** The parameters used in Equation (1).

Strength Values (*f*)		*c*_1_	*c*_2_
Concrete produced with CWG as a partial replacement for CA	*f_c_*	−0.002	−0.00005
	*f_s_*	−0.004	−0.00005
	*f_f_*	−0.006	0.00003
Concrete produced with CWG as a partial replacement for FA	*f_c_*	0.008	−0.00015
	*f_s_*	0.005	−0.00009
	*f_f_*	0.007	−0.00012

## Data Availability

Not applicable.
